# A benchmark server using high resolution protein structure data, and benchmark results for membrane helix predictions

**DOI:** 10.1186/1471-2105-14-111

**Published:** 2013-03-27

**Authors:** Emma M Rath, Dominique Tessier, Alexander A Campbell, Hong Ching Lee, Tim Werner, Noeris K Salam, Lawrence K Lee, W Bret Church

**Affiliations:** 1 Group in Biomolecular Structure and Informatics, Faculty of Pharmacy, The University of Sydney, Darlinghurst, Sydney, NSW 2006, Australia; 2 UR 1268 Biopolymères Interactions Assemblages, INRA, Nantes, 44300, France; 3 Garvan Institute of Medical Research, Darlinghurst, Sydney, NSW 2010, Australia; 4 Current address: Schrödinger Inc., 8910 University Center Lane, San Diego, CA, 92122, USA; 5 Current address: Victor Chang Cardiac Research Institute, Lowy Packer Building, 405 Liverpool St, Darlinghurst, NSW 2010, Australia

**Keywords:** Helical membrane proteins, Transmembrane helix prediction, Benchmark

## Abstract

**Background:**

Helical membrane proteins are vital for the interaction of cells with their environment. Predicting the location of membrane helices in protein amino acid sequences provides substantial understanding of their structure and function and identifies membrane proteins in sequenced genomes. Currently there is no comprehensive benchmark tool for evaluating prediction methods, and there is no publication comparing all available prediction tools. Current benchmark literature is outdated, as recently determined membrane protein structures are not included. Current literature is also limited to global assessments, as specialised benchmarks for predicting specific classes of membrane proteins were not previously carried out.

**Description:**

We present a benchmark server at http://sydney.edu.au/pharmacy/sbio/software/TMH_benchmark.shtml that uses recent high resolution protein structural data to provide a comprehensive assessment of the accuracy of existing membrane helix prediction methods. The server further allows a user to compare uploaded predictions generated by novel methods, permitting the comparison of these novel methods against all existing methods compared by the server. Benchmark metrics include sensitivity and specificity of predictions for membrane helix location and orientation, and many others. The server allows for customised evaluations such as assessing prediction method performances for specific helical membrane protein subtypes.

We report results for custom benchmarks which illustrate how the server may be used for specialised benchmarks. Which prediction method is the best performing method depends on which measure is being benchmarked. The OCTOPUS membrane helix prediction method is consistently one of the highest performing methods across all measures in the benchmarks that we performed.

**Conclusions:**

The benchmark server allows general and specialised assessment of existing and novel membrane helix prediction methods. Users can employ this benchmark server to determine the most suitable method for the type of prediction the user needs to perform, be it general whole-genome annotation or the prediction of specific types of helical membrane protein. Creators of novel prediction methods can use this benchmark server to evaluate the performance of their new methods. The benchmark server will be a valuable tool for researchers seeking to extract more sophisticated information from the large and growing protein sequence databases.

## Background

Helical transmembrane proteins are important for their involvement in cellular mechanisms, which makes them an important class of drug target. The biological function and mechanism of action of these proteins are determined by their three-dimensional (3D) structure, and membrane helices are signature elements of these structures [1]. Thus, predicting the location of membrane helices from protein sequence can provide powerful constraints for inferring 3D structure and in turn can assist to elucidate their molecular mechanisms. This endeavour is particularly important for membrane proteins, for which relatively few unique structures have been experimentally determined.

Sequencing entire organism genomes has brought an explosion in the number of available protein sequences. It is estimated that 20-30% of sequenced genomes code for helical membrane proteins [2], a figure in stark contrast to the ~1% of 3D structures determined experimentally for helical membrane proteins. This discrepancy in representations spurs development of methods for predicting membrane helices from sequence, as this permits better identification of pharmaceutically significant membrane proteins.

The topography of membrane helical segments – the description of their location in the amino acid sequence – is the focus of many current prediction methods. The most recent methods also predict whether the N-terminal and non-membrane loop segments between membrane helix segments are on the inner or outer side of the membrane – the topology of the membrane helices, which is related to their orientation in the membrane. A range of algorithmic strategies are used by prediction methods, including: amino acid hydrophobicity and other biophysical characteristics; evolutionary information in the form of multiple sequence alignments, and; machine learning strategies such as hidden Markov models, neural networks and support vector machines, which are trained on sequence data of known membrane helices.

No one membrane helix prediction method scores well for all types of scoring criteria [2]. Many methods predict the most commonly observed types of membrane helices, but many methods do not also predict the less frequently observed helices such as the half-membrane helices of the ion channels. This is because the methods have been optimised to predict only transmembrane helices that completely cross the membrane [1,2]. It is now recognised that half-membrane helices that only partially cross the membrane have importance as they constitute signature structural elements of membrane helical protein families, such as the potassium channels, aquaporins, chloride channels, the glutamate homologue transporter, and the protein conducting channel [3]. Half-membrane helices have previously been inventoried and classified as being re-entrant loops consisting of either a helix-turn-coil, coil-turn-helix or helix-turn-helix [4]. Recent x-ray crystallographic protein structures are now revealing more diversity of half-membrane helices such as: discontinuous non-re-entrant half-membrane helices joined by extended 5–7 residue loops in respiratory complex I [5]; a half-membrane helix connected at 70° by a 10-residue hinge to a membrane interface helix in the maltose transporter [6,7]; non-re-entrant half-membrane helices in the formate transporter [8] that are structurally homologous to the re-entrant aquaporin helix, and; a re-entrant partially 3_10_-helix in a photosystem I structure that is parallel to the membrane plane in place of a hairpin turn [9].

As membrane helix prediction methods are developed and improved, there is a continuing need to evaluate and compare their performances, to both aid method development and to directly evaluate method applicability. A benchmark tool for calculating and comparing accuracies of membrane helix topography and topology predictions from sequences would fill this need. Independent evaluations of existing membrane helix prediction methods have been conducted [10,11] but do not include important recent methods. The publications for the most recent prediction methods do report benchmark results of the method compared to a limited set of available prediction methods [3,4,12-16]. No benchmark has comprehensively evaluated the predictive power for specialised classes of transmembrane proteins using high resolution data of known protein topologies as the benchmark standard. A recent study by Tsirigos et. al. in 2012 [17] reports a comparison of 18 prediction methods, most of them recent methods, but does not provide the ability for users to run evaluations. Finally, since the gold standard for evaluating membrane helix prediction accuracy is to compare the predictions to known membrane helix positions in high resolution solved 3D structures, it is important that evaluations incorporate recent experimental structures. Many novel three-dimensional structures for membrane proteins were solved recently and were not available for the past evaluations.

## Construction and content

The benchmark server presents the user with options for controlling the inputs and outputs of the server. The inputs are: the prediction methods to be compared; the sequences on which the selected prediction methods operate, and; the reference helix assignments against which the helix predictions are compared. The outputs which the server can generate are the results of the benchmark defined by the inputs, or more detailed information about the inputs. Detail for all these parameters is given in the following subsections.

### Prediction methods

The server makes available a total of 52 sequence-based prediction methods, which break down to 24 *topographical* methods, 27 *topological* methods, and 1 method which seeks to predict membrane dipping re-entrant loops (TMLOOP [3]). The *topographical* methods seek to assign membrane-helix character to segments of input amino-acid sequences, whereas *topological* methods seek to assign to predicted membrane helices an orientation with respect to the membrane interfaces (inner or outer).

The available methods are those which were freely accessible to be run in batch mode. During the implementation of this server, all of the prediction methods were applied to all the sequences available on the server (which are detailed in the next section), and the results cached to speed up the user experience. When this caching was performed, the prediction methods were all run with default parameters. Methods, method types and parameters are listed in Additional file 1 Table S1.

### Sequence data

The protein data used by the server to benchmark predictions were sourced from the wwPDB [18] in February 2012 and consists of 1045 unique amino acid sequences. These 1045 sequences break down to: 481 sequences from polytopic or bitopic helical membrane proteins; 95 sequences from β-barrel membrane proteins, and; 469 sequences from soluble proteins.

Users can configure a subset of these sequences to be used in the benchmark of prediction methods according to a number of sequence attributes. These attributes are: similarity level; phylogenetic kingdom; transmembrane helix profile (bitopic or polytopic only, half-membrane helices or not); membrane protein structure family (as assigned by the Membrane Proteins of Known 3D Structure database [1]); experimental resolution; experimental method, and; year of submission.

The server defaults to including only sequences from helical membrane proteins with a similarity threshold of 30%, experimental resolution better than or equal to 3.5 Ångström, and experimental methods of x-ray diffraction or Solution NMR – a set of 392 sequences. Users may choose to include the sequences for β-barrel membrane proteins or soluble proteins in order to test the 'false positive' rate of prediction methods, which would be useful information when evaluating a prediction method's performance for genome-level scanning. Similarly, the options for selecting sequence data according to kingdom will assist users for the evaluation of genome scanning potential.

The set of sequences for helical transmembrane proteins has diversity in the types of helices it contains. Of the 392 default transmembrane protein sequences, 65 sequences contain one or more half-membrane helices or re-entrant loops in addition to transmembrane segments, and the remaining 327 sequences contain only transmembrane helices that completely cross the membrane. The one soluble protein dataset was derived from PDBselect25 [19] of March 2012, and reduced from 25% to less than 1% similarity by using psi-cd-hit [20].

The sets of sequences of varying levels of similarity were pre-computed using “algorithm 2” from [19], with similarity being evaluated using the EMBOSS [21] global alignment [22] and the EMBOSS local alignment [23]. The sets generated offer similarity levels from 20% to 100% with steps of 5%. For each kingdom the top 10% of the soluble sequences least similar to the helical membrane dataset sequences were retained for the soluble protein dataset, with similarity having been determined by the identities metric for matches having E-value less than 0.005 [24]. The structure-function family classification of sequences from helical membrane proteins is according to the Membrane Proteins of Known 3D Structure database [1]. The year of submission option allows users to include only sequences submitted after a certain date, which can aid in the detection of training bias in the prediction methods being surveyed which may have trained on the same data.

It is also possible for the user to upload their own predictions for the selection of sequences. This allows the comparison of novel prediction methods against the many existing methods. The sequences for a selection can be retrieved by choosing the appropriate output option, as described in the following sections.

### Reference helix assignments

Performance of topography and topology predictions is measured against membrane helix assignments in high resolution three-dimensional structures from the Protein Data Bank (wwPDB) [18]. The user can choose from 4 sets of reference helix assignments: OPM membrane helices; OPM *adjusted* membrane helices; PDBTM membrane helices, and; PDBTM membrane helices *and loops.* The server defaults to OPM *adjusted* membrane helices.

The helices in a solved 3D structure can deviate from the definition of a canonical helix, and the location of the membrane with respect to the helix can not be definitively determined from crystal and NMR structures. The definition of the membrane regions of proteins available in the server can be chosen from the Orientations of Proteins in Membranes (OPM) database [25] or the Protein Data Bank of Transmembrane Proteins (PDBTM) [26,27]. Manual visual comparison of the membrane helices common to structure-function families, as assigned by the Membrane Proteins of Known 3D Structure database, permitted the identification of short membrane helices that had not been identified as OPM membrane segments in some of the members of the family. The reference helix assignment dataset that includes these is referred to in the server as “OPM adjusted membrane helices” and hereafter is abbreviated to “OPM-adjusted”. PDBTM classifies short membrane helices as loop that includes the coil part of the re-entrant helix, and the server optionally allows these to be counted as membrane helices. This loop-inclusive reference helix assignment dataset is referred to as “PDBTM membrane helices and loops”. The OPM- and PDBTM-assigned membrane helices differ by an average of 2 residues per helix boundary definition.

For topology assignments, the benchmark server uses the assignments reported in OPM. The PDBTM assigns the two sides of the membrane without specifying which is inside or outside, and these assignments were compared to the OPM assignments to arrive at inside/outside topology assignments for benchmarks using PDBTM topography assignments. The term 'outside' is used to refer to the extracellular face of the membrane, and 'inside' to the other side. Three-dimensional structure determinations do not inherently determine how the protein is positioned in the membrane, making this processing necessary.

### Outputs

Once the parameters of a benchmark have been chosen, the user can select from a number of operations to perform. The default operation is to execute the benchmark and receive the benchmark results. Other operations available involve retrieving further information about the selected parameters the user has chosen for the benchmark. This further information includes the aligned predictions for the selected prediction methods and sequences, or the selected sequences in a variety of formats. These options allow the user to perform their own predictions on the chosen sets of sequence data, which can be uploaded to the server and included in benchmarking.

When a benchmark is performed, the results consist of a number of scores with differing granularity, each of which describes a different feature of prediction. The scores are divided into *topography* scores and *topology* scores.

For *topography* scores, the levels of granularity are:

1. per protein sequence accuracy, which measures the percentage of protein sequences for which all membrane helices are predicted correctly

2. per segment accuracy, which measures the performance of predicting individual helices and has two components:

2a. sensitivity, which is the percentage of reference helices which are correctly predicted

2a. specificity, which is the percentage of predicted helices which are actually in the reference helix dataset

3. helix boundary accuracy, which measures the ability of methods to correctly predict the residues where a helix begins and ends and has three variations

4. per residue accuracy, which measures the ability of methods to correctly assign specific helix characters to individual residues and has a number of variations

For *topology* scores, the levels of granularity are:

1. per protein sequence accuracy, which has two components:

1a. localisation, which measures the ability of methods to correctly assign localisation within the membrane environment to all segments of the protein chain

1a. orientation, which measures the ability of methods to correctly assign the N-terminal end of a protein chain to the correct locale in the membrane environment

2. per segment accuracy, which measures the ability of methods to correctly assign the orientation and localisation within the membrane environment to individual segments of the protein chain

3. per residue accuracy, which measures the ability of methods to correctly assign the orientation and localisation within the membrane environment to individual residues

The topography per-protein-sequence, per-segment, and per-residue measures provided by the now unavailable transmembrane helix benchmark server of [28] are included and extended in this new benchmark server to include topology and helix boundary prediction accuracy measures and Matthews Correlation Co-efficients (MCC) [29].

Some of these metrics were reported in other previous benchmarks [13-16]. Although per-residue scores are provided, it is the per-segment scores, also known as segment overlap (Sov) scores [30,31], that can be considered as the more informative metrics. This is because it is the secondary structure type (α-helical, β-barrel, or coil), position, and number of secondary membrane structure segments that characterise structure and function [1,31]. The differences in OPM and PDBTM assigned helix boundaries show that residue-level helix assignments are not unambiguously agreed. As an example of how a per-residue score can be misleading, predicting a highly α-helical protein to be entirely helical gives a high per-residue score, inflating the perceived performance of the prediction method [32]. The metrics provided by this benchmark server and their formulae are listed in Additional file 1 Table S2.

A visual comparison section displays observed versus predicted membrane helix positions and inside/outside topology for protein amino acid sequences to the amino acid level of detail, revealing problematic helices and topology segments in detail.

## Utility and discussion

The benchmark server was used to carry out benchmarks of all the transmembrane helix prediction methods available on the server, making use of the wide variety of parameters. Five major benchmarks were performed to test for different measures of accuracy: sensitivity; specificity; correctly predicted sequences; topology, and; helix boundaries. The results of these benchmark questions are presented in Table 1. To illustrate the flexibility of the server for carrying out specialised evaluations, specialised benchmarks for predictions of membrane channel helices were carried out and are reported in Table 2.

**Table 1:** Benchmark results showing prediction methods and their scores for benchmark measures

	**Benchmark measure (and subset of data used for benchmark)**										
	**Sensitivity**		**Specificity**		**Correctly predicted sequences**		**N-terminal topology**		**Non-membrane topology segments**	**Helix boundaries**	
**Prediction Method**	**(1)**	**(2)**	**(3)**	**(1)**	**(4)**	**(5)**	**(4)**	**(1)**	**(1)**	**(1)**	**(6)**
DAS-TMfilter	81	77	91	96	51	88				62	63
DAS1997 (loose)	82	78	67	89	55	72				57	64
DAS1997 (strict)	89	84	39	85	42	37				67	69
DAS2002	82	78	92	**97**	56	90				62	62
deltaG	89	87	75	96	63	76				72	68
Eisen (11,10)	59	57	11	58	10	5				31	27
Eisen (19,10)	41	36	9	51	2	1				17	14
Eisen (7,10)	74	71	13	60	16	7				45	39
ENSEMBLE (in MemPype)	81	81	90	95	52	89	68	62	80	67	62
HMM-TM	63	43	82	**97**	60	85	76	77	66	53	53
HMMTOP (in TOPCONS-single)	89	86	**95**	96	65	**94**	76	75	79	72	71
HMMTOP2	90	86	84	96	66	86	79	77	81	72	72
KyteD (11,10)	75	70	21	71	26	16				53	46
KyteD (19,10)	58	50	16	66	6	1				30	24
KyteD (7,10)	84	78	25	75	36	23				62	59
MemBrain	**94**	**93**	79	95	69	82				**80**	75
MEMSAT (in TOPCONS-single)	87	86	93	94	57	92	69	71	79	71	71
MEMSAT-SVM	**91**	88	46	97	**72**	11	88	84	83	**82**	**81**
MEMSAT3	88	88	46	96	**72**	15	**93**	**93**	**94**	50	66
OCTOPUS	**94**	**93**	83	**98**	**77**	85	**91**	**90**	**91**	**83**	**84**
OCTOPUS (in TOPCONS)	90	88	92	**97**	70	91	**89**	**88**	**86**	75	**76**
OHM (11,10)	72	65	16	69	16	10				42	35
OHM (19,10)	51	46	12	62	7	2				23	17
OHM (7,10)	83	79	22	73	23	16				53	47
PHDhtm (at PBIL)	77	69	70	94	47	74				58	56
PHDThtm (at PBIL)	82	78	93	95	48	91	54	55	69	66	67
Philius	90	86	**96**	**97**	69	**94**	79	79	83	77	73
Phobius	87	86	**95**	**97**	60	93	66	65	78	73	69
PolyPhobius	**91**	**89**	92	96	67	92	69	70	80	**78**	75
PRED-TMR	83	81	89	96	55	88				67	68
PRO-TMHMM (in TOPCONS)	90	88	**95**	**97**	66	**94**	**89**	**89**	85	72	73
PRODIV-TMHMM (in TOPCONS)	**94**	**91**	43	96	**74**	32	**89**	**89**	**89**	**78**	**78**
S-TMHMM (in TOPCONS-single)	85	85	**95**	96	57	93	81	83	82	69	68
SCAMPI	88	87	94	96	65	93	**89**	**88**	84	72	71
SCAMPI-multi (in TOPCONS)	90	88	92	**97**	70	91	**89**	**88**	**86**	75	**76**
SCAMPI-sequence (in TOPCONS)	88	87	94	96	65	93	84	84	82	72	71
SCAMPI-sequence (in TOPCONS-single)	88	87	94	96	65	**94**	84	84	82	71	72
SOSUI	82	83	93	96	49	90				64	62
SPLIT4	75	74	87	96	40	84				60	56
SVMtm	81	84	**95**	**97**	56	92				65	67
SVMtop	86	88	**95**	96	52	92	72	75	70	72	65
TMAP	84	81	76	**97**	55	77				59	52
TMHMM2	86	86	**96**	**97**	59	93	73	74	80	72	71
TMMOD	85	85	**96**	**97**	56	93	69	72	81	70	68
TMpred	86	81	64	94	52	65	61	65	69	70	67
TOPCONS	**91**	88	92	**98**	**72**	91	**89**	**88**	**87**	77	**77**
TOPCONS-single	89	87	**95**	96	66	**94**	81	80	82	75	75
TOPPRED2	85	82	62	94	55	62	73	72	73	70	69
VALPRED	76	70	61	94	45	64				42	33
VALPRED2	**93**	**90**	54	80	40	65				55	50
waveTM	88	84	79	96	52	80				62	58

Numbers in brackets refer to the subset of data used in the benchmark as specified in Table 3. The benchmark server metric used for sensitivity is “Qhtm %obs”, for specificity is “Qhtm %prd”, for correctly predicted sequences is “Qok”, for N-terminal topology is “Nterm”, for non-membrane topology segments is “Qio %obs”, and for helix boundaries is “QHb %obs”. The scores of the 5 highest scoring prediction methods are marked in bold (and if there is more than one method having the score of the 5^th^ highest scoring method then all methods having that score are marked in bold). Methods that do not predict topology do not have topology scores.

**Table 2:** Sensitivity benchmark results for predictions of families of membrane channels

	**Channel structure family**						
**Prediction Method**	**(1) FNT**	**(2) Amt/Rh**	**(3) Aquaporin**	**(4) Gap junction**	**(5) K**^**+ **^**channel**	**(6) Urea transport**	**(7) Other**
DAS1997 (strict)	**90**	**93**	**80**	100	**89**	83	**92**
HMMTOP (in TOPCONS-single)	86	99	75	**100**	80	**83**	97
HMMTOP2	86	99	75	**100**	80	**83**	97
MemBrain	**100**	93	89	**100**	92	75	92
MEMSAT (in TOPCONS-single)	86	99	84	**100**	74	**83**	95
MEMSAT-SVM	86	97	**100**	**100**	86	**83**	95
MEMSAT3	86	**100**	74	**100**	70	**83**	92
OCTOPUS	86	**100**	77	**100**	89	**83**	**100**
OCTOPUS (in TOPCONS)	86	97	75	**100**	81	**83**	97
Philius	86	**100**	75	**100**	83	75	89
PolyPhobius	86	**100**	73	**100**	86	**83**	97
PRO-TMHMM (in TOPCONS)	90	99	75	**100**	80	**83**	**100**
PRODIV-TMHMM (in TOPCONS)	86	**100**	75	**100**	91	**83**	**100**
SCAMPI-multi (in TOPCONS)	86	97	75	**100**	81	**83**	97
SVMtop	86	**100**	81	**100**	76	75	92
TMHMM2	86	99	75	**100**	75	**83**	89
TMMOD	86	**100**	75	**100**	79	75	84
TOPCONS	86	99	75	**100**	82	**83**	97
TOPCONS-single	86	99	75	**100**	76	**83**	97
VALPRED2	95	97	91	**100**	**96**	75	**100**

Each prediction method's score is given for the channel structure family benchmark data subset as specified in Table 4. The benchmark server metric used is “Qhtm %obs”. The highest score for each family is marked in bold. Results for prediction methods that did not obtain a highest score for any of these specialised channel benchmarks except the Gap Junction benchmark (for which all methods except OPM (19,0) scored 100%) have been omitted.

These benchmarks were all performed using the standard interface to the server and can be performed by any user.

### Benchmarking considerations

Using the server's data selection features, specifically appropriate benchmark data subsets were chosen to illustrate the extent of differences in prediction accuracy for the different benchmark metrics involved in each benchmark question. These data subsets are specified in Table 3. For sensitivity benchmarks, only membrane sequences containing at least one membrane helix were included in the benchmark, so as not to dilute the differences in sensitivity accuracy with statistics for sequences not containing any membrane helices. However, when performing **s**pecificity benchmarks, it is highly relevant to include sequences that do not contain membrane helices so that false positive rates may be assessed, and so specificity benchmarks included such sequences. Specificity benchmarks were also carried out on datasets where the only included membrane sequences were those containing at least one membrane helix.

**Table 3:** Characteristics of the subsets of data used for the benchmarks reported in this paper

	**Characteristics of the specialised data subsets used for the benchmarks**									
**Identifier for subsets of data used in benchmarks reported in this paper**	**TMH**	**½MH**	**BB**	**Solb**	**All years**	**2008…**	**OPM**	**PDBTM**	**#seqs**	**#MHs**
(1) TMH_1/2MH_OPM	Y	Y			Y		Y		101	483
(2) TMH_1/2MH_2008_OPM	Y	Y				Y	Y		24	191
(3) TMH_1/2MH_BB_SOLB_OPM	Y	Y	Y	Y	Y		Y		599	483
(4) TMH_OPM	Y				Y		Y		86	372
(5) TMH_BB_SOLB_OPM	Y		Y	Y	Y		Y		584	372
(6) TMH_PDBTM	Y				Y			Y	86	464

All these data subsets were restricted to sequences having less than 30% similarity to each other with similarity having been measured by EMBOSS global sequence alignment. Other parameters used to build the data subsets are specified by ticks in the columns. For parameters not specified here the benchmark server default values were used. Legend : TMH : transmembrane helices; ½MH : half-membrane helices; BB : membrane β-barrels; Solb : soluble proteins; All years : sequences used in the benchmark were not restricted by date that the PDB model was made available; 2008 : benchmark was carried out restricting sequences to those belonging to PDB structures deposited 2008 or after and not having any structures of similar sequence deposited before 2008. OPM : benchmark was carried out using OPM-adjusted membrane helix assigments. PDBTM : benchmark was carried out using PDBTM membrane helix assignments without including segments assigned as loops. #seqs : total number of sequences; #MHs : total number of membrane helices.

For topology assessments, benchmarks of datasets both containing and excluding half-membrane helices were performed, because difficulties in predicting half-membrane helices can adversely affect the topology performance. For helix boundaries assessments, benchmarks were carried out separately for the benchmark data using OPM-defined membrane helices versus that of PDBTM, because these two definitions do not assign helix boundaries identically. Apart from this assessment, all benchmarks were carried out using only the OPM-adjusted membrane helix assignments. All benchmarks were carried out on a data subset where member sequences were restricted to less than 30% similarity to other sequences in the subset, with similarity having been measured by EMBOSS global sequence alignment. Unless otherwise specified, the default benchmark server parameters were used.

For the specialised benchmarks on membrane channel predictions, the benchmark data were restricted to only sequences belonging to specific membrane protein structure families, as specified in Table 4. As there are not many benchmark sequences available in the server for each family of membrane channels, benchmark dataset sequences were not restricted by similarity to each other. For all other parameters the benchmark server defaults were used.

**Table 4:** Counts of sequences and membrane helices of the data subsets used for the specialised benchmarks for channels reported in this paper

**Channel structure family**	**Abbreviated channel structure family**	**#seqs**	**#TMH**	**#½MH**	**Total #MH**
Channels: Formate Nitrate Transporter (FNT) Family	(1) FNT	3	19	3	22
Channels: Amt/Rh proteins	(2) Amt/Rh	6	67	0	67
Channels: Aquaporins and Glyceroporins	(3) Aquaporin	12	72	24	96
Channels: Gap Junctions	(4) Jap junction	1	4	0	4
Channels: Potassium and Sodium Ion-Selective	(5) K^+^ channel	25	80	25	105
Channels: Urea Transporters	(6) Urea transport	1	10	2	12
Channels: Other Ion Channels	(7) Other	14	37	0	37

The benchmark server's membrane protein structure family selections were used to restrict each benchmark to a membrane channel structure family. There was no restriction on similarity (the benchmark server's similarity level was set to 100%), and for all other options the server's default parameters were used. Legend : #seqs : number of sequences; #TMH : number of transmembrane helices that cross the membrane; #½MH : number of half-membrane helices; Total #MH : total number of membrane helices.

The prediction methods were all used with their default parameter settings, and so the benchmark results are presented with the caveat that the methods may perform better when their parameters have been optimised for the specific prediction question being judged by the benchmark.

### Sensitivity

The highest scoring methods for sensitivity are prediction methods using a range of different algorithms and information, such as machine learning, biophysical properties, sequence alignments and consensus, with no one strategy showing superiority at predicting membrane helices with sensitivity. These benchmarks also demonstrate that a consensus method (TOPCONS) [33] scores lower than the highest scoring method used to compile its consensus (PRODIV-TMHMM) [15,33].

To investigate how well prediction methods perform on data that was not used to calibrate the method, the benchmarks were repeated restricting benchmark data to the wwPDB structures that were released in 2008 or after and not having any similar sequences in the wwPDB before then. The resulting scores were on average 4% lower. This result, and the observation that older machine learning methods do not perform as well as newer machine learning methods in the overall sensitivity benchmarks, suggests that machine learning method prediction sensitivities might benefit from the methods being retrained on the latest available data, and demonstrates that prediction methods generally do not perform quite as well for sensitivity on data that is not similar to that which was used for the method creation. Machine learning prediction methods are highly represented in the set of top scores of sensitivity benchmarks, with 5 out of 7 of the highest scoring methods reported in Table 1 being machine learning methods.

Purely biophysical methods do appear as top scoring sensitivity methods – VALPRED2 [34] is in the list of top 5 methods, and SCAMPI-multi [33,35] is in the top 10 methods. This may indicate that biophysical based methods developed in the future using knowledge about the forces that drive the membrane helix formation process have the potential to give superior prediction sensitivity performance. However, the simpler biophysical based methods using scores based only on hydrophobicity perform significantly worse than the other methods.

### Specificity

The highly sensitive prediction methods, with the exception of VALPRED2, have high specificity scores of 95% and above for the benchmark on membrane helical sequences, showing the welcome result that sensitivity has not come at the expense of generating many false positives. The list of the prediction methods obtaining the highest specificity score changes completely when the β-barrel and soluble sequences are included with the membrane helical sequences in the benchmark. This indicates that the choice of prediction method, having the best specificity, to employ should depend on whether the predictions are on proteins that are already known to be helical membrane proteins – as is the case of detailed investigations of specific membrane proteins – or are known to contain soluble proteins too – as is the case for genome annotation.

### Correctly predicted sequences

The highest performing method in benchmarks for correctly predicting all and only all observed membrane helices in helical membrane protein sequences is OCTOPUS [16,36], and as was the case with specificity, the list of best methods changes completely when β-barrel membrane and soluble protein sequences are included in the benchmark, with OCTOPUS dropping to 28^th^ place. These benchmark results suggest a two-pronged approach is appropriate for the task of automated genome annotation, which requires correct sensitivity and specificity. First use a prediction method that exhibits high specificity for discriminating between membrane helical and non membrane helical sequences to identify sequences having membrane helices, and for those sequences, use OCTOPUS predictions for the actual membrane helix annotation, thus avoiding the false positive predictions of OCTOPUS for non membrane helical proteins.

### Topology

The OCTOPUS and MEMSAT-SVM [14] prediction methods predict re-entrant segments and adjust the topology prediction accordingly by predicting both sides of the re-entrant segment to be on the same side instead of alternating inside/outside. However, the MEMSAT3 [37,38] method scores better, and other methods score almost as well as OCTOPUS, and better than MEMSAT-SVM, for correctly assigning inside/outside topology – even though they don't predict re-entrant loops. This is due to those methods not considering re-entrant loops altogether, thus removing the possibility of putting the alternating inside/outside topology prediction out of order.

### Membrane helix boundaries

OCTOPUS is consistently best in benchmarks for predicting helix boundaries to the residue regardless of whether the OPM or PDBTM data are used as the benchmark data, even though OPM and PDBTM do not always assign the same helix boundaries as each other.

### Specialised benchmarks for channels

Benchmarks of membrane helix predictions for channel families were performed. The results are shown in Table 2 and suggest which prediction methods are best for predicting membrane helices in the different channel families, and show that not all methods predict these signature membrane helices.

## Conclusions

This benchmark server for assessing predictions of membrane helices from sequence contains recent high resolution 3D structure data and thus provides the most accurate benchmark currently possible. Prediction of membrane helices from sequences continues to be a valuable activity and it is appropriate that recently available 3D structure data be used for the benchmarking of such predictions. We reported the results of various analytical benchmarks carried out by this server.

The benchmark server provides sub-categorisations, combinations, and customisation of the benchmark data, providing the ability to customise benchmarks for specific benchmark purposes. This allows users to assess which are the best prediction algorithms for varied applications. The data-selection capabilities, coupled with the ability to enter and benchmark the results of novel prediction methods, permit the tuning and assessment of novel prediction algorithms. The use of this server provided insights into currently available prediction methods. For example, benchmark results from this server suggest a two-pronged approach for membrane helix genome annotation, given the discovery that the prediction methods most sensitive to clean data are usually insensitive to 'messy' data, which would be the case with genomes. The server also suggests the possibility of training bias in the machine learning methods surveyed.

We present this benchmark server as a tool for comparing current membrane helix prediction methods, and for comparing novel methods against current methods so that one might meaningfully evaluate their performance and suitability to a variety of bioinformatic tasks.

## Availability and requirements

Project name: Benchmark of Membrane Helix Predictions from Sequence

Project home page : http://sydney.edu.au/pharmacy/sbio/software/TMH_benchmark.shtml

Operating systems : The user accesses the benchmark server through a standard Internet web browser. The server runs on a Linux platform.

Programming language : Perl

Other requirements : There are no other requirements for the user other than a computer Internet web browser.

License : The Perl software of the benchmark server will be released under an open source software license such as GNU General Public License or Creative Commons license for Free Software Foundation's GNU General Public License at creativecommons.org.

Restrictions to use by non-academics: There are no restrictions on use of this benchmark server.

## Abbreviations

MCC: Matthews Correlation Co-efficient [29]; OPM: Orientations of Proteins in Membranes database [25]; PDBTM: PDBTM Protein Data Bank of Transmembrane Proteins [26,27]; Sov: Segment overlap [30,31]; wwPDB: Protein Data Bank; 3D: Three-dimensional.

## Competing interests

The authors declare that they have no competing interests.

## Authors’ contributions

EMR : Conception, design, coding, and testing of benchmark server code and dataset preparation. Writing of manuscript. Porting, by rewriting in Perl, and testing of the VALPRED/REPIMPS membrane helix prediction tools to a server and batch platform. Modification of VALPRED algorithm to produce VALPRED2 for prediction of certain types of helices. DT : Conception, design, and testing of benchmark server and dataset preparation. Writing of manuscript. AAC : Conception of benchmark server and dataset preparation. Writing and structure of manuscript. HCL : Conception of benchmark server and dataset preparation. Writing of manuscript. TW : Dataset preparation. Writing of manuscript. NKS : Writing, testing and benchmarking of VALPRED/REPIMPS. Writing of manuscript. LKL : Writing, testing and benchmarking of VALPRED/REPIMPS. Writing of manuscript. WBC : Conception and design of benchmark server and dataset preparation. Conception, design, coding and testing of the VALPRED and REPIMPS membrane helix prediction tools. Writing of manuscript. All authors read and approved the final manuscript.

## Supplementary Material

Additional file 1: Table S1A Prediction methods benchmarked in the benchmark server. **Table S1B. **Default benchmark parameters that can be adjusted by user. **Table S2. **Benchmark metrics and their formulae. Click here for file
