# Supplementary material for: A benchmark server using high resolution protein structure data, and benchmark results for membrane helix predictions
Source: BMC Bioinformatics. 2013 Mar 27;14:111. doi: 10.1186/1471-2105-14-111 (PMC3620685; doi:10.1186/1471-2105-14-111)
Supplement: Additional file 1: Table S1A — Prediction methods benchmarked in the benchmark server. Table S1B. Default benchmark parameters that can be adjusted by user. Table S2. Benchmark metrics and their formulae. [file 1471-2105-14-111-S1.doc]

### Supplementary Information

***Supplementary Table S1A. Prediction methods benchmarked in the benchmark server.***

| **Method name**  **Truncated method name for visual alignment report** | **Website where prediction method's software was obtained or executed**  **Method's citations** |
| --- | --- |
| DAS-TMfilter  DASTMfilt | http://www.enzim.hu/DAS/DAS.html  Cserzo M, Eisenhaber F, Eisenhaber B, Simon I (2004) TM or not TM: transmembrane protein prediction with low false positive rate using DAS-TMfilter. Bioinformatics, 20, 1, 136-137.  (uses sequence alignment of membrane protein sequences and hydropathy dot-plots) |
| DAS2002  DAS2002 | http://mendel.imp.ac.at/DAS/  Cserzo M, Eisenhaber F, Eisenhaber B, Simon I (2002) On filtering false positive transmembrane protein predictions. Protein Engineering, 15, 745-752.  (uses sequence alignment of membrane protein sequences and hydropathy dot-plots) |
| DAS1997 (loose)  DAS1997l | http://www.sbc.su.se/~miklos/DAS/ cutoff=1.7  Cserzo M, Wallin E, Simon I, von Heijne G, Elofsson A (1997) Prediction of transmembrane alpha-helices in procariotic membrane proteins: the Dense Alignment Surface method. Protein Engineering, 10, 673-676.  (uses sequence alignment of membrane protein sequences and hydropathy dot-plots) |
| DAS1997 (strict)  DAS1997s | http://www.sbc.su.se/~miklos/DAS/ cutoff=2.2  Cserzo M, Wallin E, Simon I, von Heijne G, Elofsson A (1997) Prediction of transmembrane alpha-helices in procariotic membrane proteins: the Dense Alignment Surface method. Protein Engineering, 10, 673-676.  (uses sequence alignment of membrane protein sequences and hydropathy dot-plots) |
| deltaG  deltaG | http://www.cbr.su.se/DGpred/  Hessa T, Meindl-Beinker N, Bernsel A, Kim J, Sato Y, Lerch M, Lundin C, Nilsson I, White SH, von Heijne G (2007) Molecular code for transmembrane-helix recognition by the Sec61 translocon. Nature, 450, 1026-1030.  (uses experimentally derived biophysical residue free energy values for insertion into membranes) |
| Eisenberg (7,10)  Eisen(7) | ftp://emboss.open-bio.org/pub/EMBOSS/EMBOSS-6.4.0.tar.gz EMBOSS 6.4.0 pepinfo method=Eisenberg window=7 cutoff=10, minimum helix length of 10  Eisenberg D, Weiss RM, Terwilliger TC (1982) The helical hydrophobic moment: a measure of the amphiphilicity of a helix. Nature, 299, 371-374. Rice P, Longden I, Bleasby A (2000) EMBOSS: the European Molecular Biology open software suite. Trends in Genetics, 16, 276-277.  (uses biophysical residue hydropathy values) |
| Eisenberg (11,10)  Eisen(11) | ftp://emboss.open-bio.org/pub/EMBOSS/EMBOSS-6.4.0.tar.gz EMBOSS 6.4.0 pepinfo method=Eisenberg window=11 cutoff=10, minimum helix length of 10  Eisenberg D, Weiss RM, Terwilliger TC (1982) The helical hydrophobic moment: a measure of the amphiphilicity of a helix. Nature, 299, 371-374. Rice P, Longden I, Bleasby A (2000) EMBOSS: the European Molecular Biology open software suite. Trends in Genetics, 16, 276-277.  (uses biophysical residue hydropathy values) |
| Eisenberg (19,10)  Eisen(19) | ftp://emboss.open-bio.org/pub/EMBOSS/EMBOSS-6.4.0.tar.gz EMBOSS 6.4.0 pepinfo method=Eisenberg window=19 cutoff=10, minimum helix length of 10  Eisenberg D, Weiss RM, Terwilliger TC (1982) The helical hydrophobic moment: a measure of the amphiphilicity of a helix. Nature, 299, 371-374. Rice P, Longden I, Bleasby A (2000) EMBOSS: the European Molecular Biology open software suite. Trends in Genetics, 16, 276-277.  (uses biophysical residue hydropathy values) |
| ENSEMBLE (in MemPype)  ENSEMBLE | http://mu2py.biocomp.unibo.it/mempype  Martelli PL, Fariselli P, Casadio R. (2003) An ENSEMBLE machine learning approach for the prediction of all-alpha membrane proteins. Bioinformatics, 19, Suppl 1, i205-11.  Pierleoni A, Indio V, Savojardo C, Fariselli P, Martelli PL, Casadio R. (2011) MemPype: a pipeline for the annotation of eukaryotic membrane proteins. Nucleic Acids Res, 39(Web Server issue), W375-80.  (uses neural network (NN) and hidden Markov models (HMM) trained on membrane protein sequences) |
| HMM-TM  HMM-TM | http://bioinformatics.biol.uoa.gr/HMM-TM/  Bagos PG, Liakopoulos TD, Hamodrakas SJ (2006) Algorithms for incorporating prior topological information in HMMs: application to transmembrane proteins. BMC Bioinformatics, 5, 7, 189.  (uses hidden Markov model (HMM) trained on membrane protein sequences) |
| HMMTOP2  HMMTOP2 | http://www.enzim.hu/hmmtop/  Tusnády GE, Simon I (1998) Principles governing amino acid composition of integral membrane proteins: applications to topology prediction. Journal of Molecular Biology, 283, 489-506. Tusnády GE, Simon I (2001) The HMMTOP transmembrane topology prediction server. Bioinformatics, 17, 849-850.  (uses hidden Markov model (HMM) trained on membrane protein sequences) |
| HMMTOP (in TOPCONS-single)  HMMTOPs | http://single.topcons.net  Tusnády GE, Simon I (1998) Principles governing amino acid composition of integral membrane proteins: applications to topology prediction. Journal of Molecular Biology, 283, 489-506. Tusnády GE, Simon I (2001) The HMMTOP transmembrane topology prediction server. Bioinformatics 17, 849-850. Hennerdal A, Elofsson A (2011) Rapid membrane protein topology prediction. Bioinformatics 27, 9, 1322-1323.  (uses hidden Markov model (HMM) trained on membrane protein sequences) |
| Kyte-Doolittle (7,10)  KyteD(7) | ftp://emboss.open-bio.org/pub/EMBOSS/EMBOSS-6.4.0.tar.gz EMBOSS 6.4.0 pepinfo method=Kyte-Doolittle window=7 cutoff=10, minimum helix length of 10  Kyte J, Doolittle RF (1982) A simple method for displaying the hydropathic character of a protein. Journal of Molecular Biology, 157, 105-132. Rice P, Longden I, Bleasby A (2000) EMBOSS: the European Molecular Biology open software suite. Trends in Genetics, 16, 276-277.  (uses biophysical residue hydropathy values) |
| Kyte-Doolittle (11,10)  KyteD(11) | ftp://emboss.open-bio.org/pub/EMBOSS/EMBOSS-6.4.0.tar.gz EMBOSS 6.4.0 pepinfo method=Kyte-Doolittle window=11 cutoff=10, minimum helix length of 10  Kyte J, Doolittle RF (1982) A simple method for displaying the hydropathic character of a protein. Journal of Molecular Biology, 157, 105-132. Rice P, Longden I, Bleasby A (2000) EMBOSS: the European Molecular Biology open software suite. Trends in Genetics, 16, 276-277.  (uses biophysical residue hydropathy values) |
| Kyte-Doolittle (19,10)  KyteD(19) | ftp://emboss.open-bio.org/pub/EMBOSS/EMBOSS-6.4.0.tar.gz EMBOSS 6.4.0 pepinfo method=Kyte-Doolittle window=19 cutoff=10, minimum helix length of 10  Kyte J, Doolittle RF (1982) A simple method for displaying the hydropathic character of a protein. Journal of Molecular Biology, 157, 105-132. Rice P, Longden I, Bleasby A (2000) EMBOSS: the European Molecular Biology open software suite. Trends in Genetics, 16, 276-277.  (uses biophysical residue hydropathy values) |
| MemBrain  MemBrain | http://chou.med.harvard.edu/bioinf/MemBrain  Shen H, Chou JJ (2008) MemBrain: improving the accuracy of predicting transmembrane helices. PLoS One. 11, 3, e2399.  (uses sequence alignment of membrane protein sequences and machine learning trained on membrane protein sequences) |
| MEMSAT-SVM  MEMSATSVM | http://bioinf.cs.ucl.ac.uk/psipred/?program=svmmemsat  Nugent T, Jones DT (2009) Transmembrane protein topology prediction using support vector machines. BMC Bioinformatics, 10, 159.  (uses support vector machine (SVM) trained on membrane protein sequences) |
| MEMSAT3  MEMSAT3 | http://bioinf.cs.ucl.ac.uk/psipred/?program=svmmemsat  Jones DT (2007) Improving the accuracy of transmembrane protein topology prediction using evolutionary information. Bioinformatics, 23, 5, 538-544. Jones DT, Taylor WR, Thornton JM (1994) A model recognition approach to the prediction of all-helical membrane protein structure and topology. Biochemistry, 33, 10, 3038-3049.  (uses artificial neural network (NN) trained on membrane protein sequences) |
| MEMSAT (in TOPCONS-single)  MEMSATs | http://single.topcons.net  Jones DT, Taylor WR, Thornton JM (1994) A model recognition approach to the prediction of all-helical membrane protein structure and topology. Biochemistry, 33, 3038-3049. Hennerdal A, Elofsson A (2011) Rapid membrane protein topology prediction. Bioinformatics 27, 9, 1322-1323.  (uses artificial neural network (NN) trained on membrane protein sequences) |
| OCTOPUS  OCTOPUS | http://octopus.cbr.su.se/  Viklund H, Elofsson A (2008) OCTOPUS: improving topology prediction by two-track ANN-based preference scores and an extended topological grammar. Bioinformatics, 24, 15, 1662-1668. Viklund H, Bernsel A, Skwark M, Elofsson A (2008) SPOCTOPUS: a combined predictor of signal peptides and membrane protein topology. Bioinformatics, 24, 24, 2928-2929.  (uses hidden Markov models (HMM) and artificial neural networks (NN) trained on membrane protein sequences) |
| OCTOPUS (in TOPCONS)  OCTOPUSt | http://topcons.cbr.su.se/  Viklund H, Elofsson A (2008) OCTOPUS: improving topology prediction by two-track ANN-based preference scores and an extended topological grammar. Bioinformatics, 24, 15, 1662-1668. Viklund H, Bernsel A, Skwark M, Elofsson A (2008) SPOCTOPUS: a combined predictor of signal peptides and membrane protein topology. Bioinformatics, 24, 24, 2928-2929. Bernsel A, Viklund H, Hennerdal A, Elofsson A (2009) TOPCONS: consensus prediction of membrane protein topology. Nucleic Acids Research, Web Server Issue 37, W465-W468.  (uses hidden Markov models (HMM) and artificial neural networks (NN) trained on membrane protein sequences) |
| OHM (7,10)  OHM(7) | ftp://emboss.open-bio.org/pub/EMBOSS/EMBOSS-6.4.0.tar.gz EMBOSS 6.4.0 pepinfo method=OHM window=7 cutoff=10, minimum helix length of 10  Sweet RM, Eisenberg D (1983) Correlation of sequence hydrophobicities measures similarity in three-dimensional protein structure. Journal of Molecular Biology, 171, 479-488. Rice P, Longden I, Bleasby A (2000) EMBOSS: the European Molecular Biology open software suite. Trends in Genetics, 16, 276-277.  (uses biophysical residue hydropathy values) |
| OHM (11,10)  OHM(11) | ftp://emboss.open-bio.org/pub/EMBOSS/EMBOSS-6.4.0.tar.gz EMBOSS 6.4.0 pepinfo method=OHM window=11 cutoff=10, minimum helix length of 10  Sweet RM, Eisenberg D (1983) Correlation of sequence hydrophobicities measures similarity in three-dimensional protein structure. Journal of Molecular Biology, 171, 479-488. Rice P, Longden I, Bleasby A (2000) EMBOSS: the European Molecular Biology open software suite. Trends in Genetics, 16, 276-277.  (uses biophysical residue hydropathy values) |
| OHM (19,10)  OHM(19) | ftp://emboss.open-bio.org/pub/EMBOSS/EMBOSS-6.4.0.tar.gz EMBOSS 6.4.0 pepinfo method=OHM window=19 cutoff=10, minimum helix length of 10  Sweet RM, Eisenberg D (1983) Correlation of sequence hydrophobicities measures similarity in three-dimensional protein structure. Journal of Molecular Biology, 171, 479-488. Rice P, Longden I, Bleasby A (2000) EMBOSS: the European Molecular Biology open software suite. Trends in Genetics, 16, 276-277.  (uses biophysical residue hydropathy values) |
| PHDhtm (at PBIL)  PHDhtm | http://npsa-pbil.ibcp.fr/cgi-bin/npsa_automat.pl?page=/NPSA/npsa_htm.html  Rost B, Sander C (1994) Combining evolutionary information and neural networks to predict protein secondary structure. Proteins, 19, 55-72. Rost B, Sander C (1993) Prediction of protein secondary structure at better than 70% accuracy. J. Mol. Biol., 232, 584-599. Rost B, Sander C (1993) Improved prediction of protein secondary structure by use of sequence profiles and neural networks. Proc. Natl. Acad. Sci. U.S.A., 90, 7558-7562.  Rost B, Casadio R, Fariselli P, Sander C (1995) Transmembrane helices predicted at 95% accuracy. Protein Sci, 4, 3, 521-533. Combet C, Blanchet C, Geourjon C, Deléage G (2000) NPS@: Network Protein Sequence Analysis. TIBS 2000, 3, 291, 147-150.  (uses sequence alignment of membrane protein sequences and artificial neural network (NN) trained on membrane protein sequences) |
| PHDThtm (at PBIL)  PHDThtm | http://npsa-pbil.ibcp.fr/cgi-bin/npsa_automat.pl?page=/NPSA/npsa_htm.html  Rost B, Sander C (1994) Combining evolutionary information and neural networks to predict protein secondary structure. Proteins, 19, 55-72. Rost B, Sander C (1993) Prediction of protein secondary structure at better than 70% accuracy. J. Mol. Biol., 232, 584-599. Rost B, Sander C (1993) Improved prediction of protein secondary structure by use of sequence profiles and neural networks. Proc. Natl. Acad. Sci. U.S.A., 90, 7558-7562.  Rost B, Casadio R, Fariselli P, Sander C (1995) Transmembrane helices predicted at 95% accuracy. Protein Sci, 4, 3, 521-533. Combet C, Blanchet C, Geourjon C, Deléage G (2000) NPS@: Network Protein Sequence Analysis. TIBS 2000, 3, 291, 147-150.  (uses sequence alignment of membrane protein sequences and artificial neural network (NN) trained on membrane protein sequences) |
| Philius  Philius | http://www.yeastrc.org/philius/pages/philius/uploadFASTA.jsp http://www.yeastrc.org/philius/pages/philius/runPhilius.jsp  Reynolds SM, Käll L, Riffle ME, Bilmes JA, Noble WS (2008) Transmembrane topology and signal peptide prediction using dynamic bayesian networks. PLoS Comput Biol, 4, 11, e1000213.  (uses dynamic Bayesian network trained on membrane protein sequences) |
| Phobius  Phobius | http://phobius.cgb.ki.se  Käll L, Krogh A, Sonnhammer EL (2004) A Combined Transmembrane Topology and Signal Peptide Prediction Method. J Mol Biol, 14, 5, 1027-1036. Käll L, Krogh A, Sonnhammer EL (2007) Advantages of combined transmembrane topology and signal peptide prediction--the Phobius web server. Nucleic Acids Res, 35, Web Server issue, W429-432.  (uses hidden Markov model (HMM) trained on membrane protein sequences) |
| PolyPhobius  PolyPhobs | http://phobius.sbc.su.se/poly.html  Käll L, Krogh A, Sonnhammer EL (2005) An HMM posterior decoder for sequence feature prediction that includes homology information. Bioinformatics, 21, Suppl 1, i251-257. Käll L, Krogh A, Sonnhammer EL (2007) Advantages of combined transmembrane topology and signal peptide prediction--the Phobius web server. Nucleic Acids Res, 35, Web Server issue, W429-432.  (uses hidden Markov model (HMM) trained on membrane protein sequences and sequence alignment of membrane protein sequences) |
| PRED-TMR  PRED-TMR | http://athina.biol.uoa.gr/PRED-TMR/input.html  Pasquier C, Promponas VJ, Palaios GA, Hamodrakas JS, Hamodrakas SJ (1999) A novel method for predicting transmembrane segments in proteins based on a statistical analysis of the SwissProt database: the PRED-TMR algorithm. Protein Engineering, 12, 381-385.  (uses statistical analysis of protein database) |
| PRO-TMHMM (in TOPCONS)  PRO | http://topcons.cbr.su.se/  Viklund H, Elofsson A (2004) Best alpha-helical transmembrane protein topology predictions are achieved using hidden Markov models and evolutionary information. Protein Sci, 13, 7, 1908-1917. Bernsel A, Viklund H, Hennerdal A, Elofsson A (2009) TOPCONS: consensus prediction of membrane protein topology. Nucleic Acids Research, Web Server Issue 37, W465-W468.  (uses sequence alignment of membrane protein sequences and hidden Markov model (HMM) trained on membrane protein sequences) |
| PRODIV-TMHMM (in TOPCONS)  PRODIV | http://topcons.cbr.su.se/  Viklund H, Elofsson A (2004) Best alpha-helical transmembrane protein topology predictions are achieved using hidden Markov models and evolutionary information. Protein Sci, 13, 7, 1908-1917. Bernsel A, Viklund H, Hennerdal A, Elofsson A (2009) TOPCONS: consensus prediction of membrane protein topology. Nucleic Acids Research, Web Server Issue 37, W465-W468.  (uses sequence alignment of membrane protein sequences and hidden Markov model (HMM) trained on membrane protein sequences) |
| SCAMPI  SCAMPI | http://scampi.cbr.su.se/  Bernsel A, Viklund H, Falk J, Lindahl E, von Heijne G, Elofsson A (2008) Prediction of membrane-protein topology from first principles. Proc. Natl. Acad. Sci. USA. 105, 7177-7181.  (uses biophysical residue hydropathy values and principles of translocon functioning) |
| SCAMPI-multi (in TOPCONS)  SCAMPImuT | http://topcons.cbr.su.se/  Bernsel A, Viklund H, Falk J, Lindahl E, von Heijne G, Elofsson A (2008) Prediction of membrane-protein topology from first principles. Proc. Natl. Acad. Sci. USA. 105, 7177-7181. Bernsel A, Viklund H, Hennerdal A, Elofsson A (2009) TOPCONS: consensus prediction of membrane protein topology. Nucleic Acids Research, Web Server Issue 37, W465-W468.  (uses sequence alignment of membrane protein sequences, biophysical residue hydropathy values and principles of translocon functioning) |
| SCAMPI-single (in TOPCONS-single)  SCAMPIsiS | http://single.topcons.net  Bernsel A, Viklund H, Falk J, Lindahl E, von Heijne G, Elofsson A (2008) Prediction of membrane-protein topology from first principles. Proc. Natl. Acad. Sci. USA. 105, 7177-7181. Hennerdal A, Elofsson A (2011) Rapid membrane protein topology prediction. Bioinformatics 27, 9, 1322-1323.  (uses biophysical residue hydropathy values and principles of translocon functioning) |
| SCAMPI-single (in TOPCONS)  SCAMPIsiT | http://topcons.cbr.su.se/  Bernsel A, Viklund H, Falk J, Lindahl E, von Heijne G, Elofsson A (2008) Prediction of membrane-protein topology from first principles. Proc. Natl. Acad. Sci. USA. 105, 7177-7181. Hennerdal A, Elofsson A (2011) Rapid membrane protein topology prediction. Bioinformatics 27, 9, 1322-1323. Bernsel A, Viklund H, Hennerdal A, Elofsson A (2009) TOPCONS: consensus prediction of membrane protein topology. Nucleic Acids Research, Web Server Issue 37, W465-W468.  (uses biophysical residue hydropathy values and principles of translocon functioning) |
| SOSUI  SOSUI | http://bp.nuap.nagoya-u.ac.jp/sosui/sosuiG/sosuigsubmit.html  Hirokawa T, Boon-Chieng S, Mitaku S (1998) SOSUI: classification and secondary structure prediction system for membrane proteins. Bioinformatics, 14, 378-379. Mitaku S, Hirokawa T (1999) Physicochemical factors for discriminating between soluble and membrane proteins: hydrophobicity of helical segments and protein length. Protein Engineering, 11, 953-957. Mitaku S, Hirokawa T, Tsuji T (2002) Amphiphilicity index of polar amino acids as an aid in the characterization of amino acid preference at membrane-water interfaces. Bioinformatics, 18, 608-616.  (uses biophysical residue hydropathy values and other physico-chemical properties) |
| SPLIT4  SPLIT4 | http://split.pmfst.hr/split/4/  Juretic D, Zoranic L, Zucic D (2002) Basic charge clusters and predictions of membrane protein topology. Journal of Chemical Information and Modeling, 42, 620-632.  (uses biophysical residue hydropathy values and other physico-chemical properties) |
| SVMtm  SVMtm | http://ccb.imb.uq.edu.au/svmtm/  Yuan Z, Mattick JS, Teasdale RD (2004) SVMtm: Support vector machines to predict transmembrane segments. Journal of Computational Chemistry, 25, 632-636.  (uses support vector machine (SVM) trained on membrane protein sequences) |
| SVMtop  SVMtop | http://bio-cluster.iis.sinica.edu.tw/~bioapp/SVMtop/  Lo A, Chiu HS, Sung TY, Lyu PC, Hsu WL (2008) Enhanced membrane protein topology prediction using a hierarchical classification method and a new scoring function. J Proteome Res. 7, 2, 487-496.  (uses support vector machine (SVM) trained on membrane protein sequences) |
| EMBOSS TMAP  TMAP | ftp://emboss.open-bio.org/pub/EMBOSS/EMBOSS-6.4.0.tar.gz (TMAP was run without any sequence alignment inputs.)  Persson B, Argos P (1994) Prediction of transmembrane segments in proteins utilising multiple sequence alignments. Journal of Molecular Biology, 237, 182-192. Persson B, Argos P (1996) Topology prediction of membrane proteins. Protein Science, 5, 363-371.  (uses sequence alignment of membrane protein sequences) |
| TMHMM Server v. 2.0  TMHMM2 | http://www.cbs.dtu.dk/services/TMHMM/  Sonnhammer EL, von Heijne G, Krogh A (1998) A hidden Markov model for predicting transemembrane helices in protein sequences. Proceeding of Sixth International Conference on Intelligent Systems for Molecular Biology, Vol. 5, AAAI/MIT Press, Menlo Park, CA, pp. 175-182. Krogh A, Larsson B, von Heijne G, Sonnhammer EL (2001) Predicting transmembrane protein topology with a hidden Markov model: application to complete genomes. Journal of Molecular Biology, 305, 567-580.  (uses hidden Markov model (HMM) trained on membrane protein sequences) |
| S-TMHMM (in TOPCONS-single)  STMHMMs | http://single.topcons.net  Viklund H, Elofsson A (2004) Best alpha-helical transmembrane protein topology predictions are achieved using hidden Markov models and evolutionary information. Protein Sci, 13, 7, 1908-1917.  (uses hidden Markov model (HMM) trained on membrane protein sequences) |
| TMLOOP  TMLOOP | http://membraneproteins.swan.ac.uk/TMLOOP  Lasso G, Antoniw JF, Mullins JGL (2006) A combinatorial pattern discovery approach for the prediction of membrane dipping (re-entrant) loops. Bioinformatics 22, 14, e290-e297.  (uses combinatorial pattern discovery trained on membrane protein sequences) |
| TMMOD  TMMOD | http://liao.cis.udel.edu/website/servers/TMMOD/  Kahsay RY, Gao G, Liao L (2005) An improved hidden Markov model for transmembrane protein detection and topology prediction and its applications to complete genomes. Bioinformatics, 21, 9, 1853-1858.  (uses hidden Markov model (HMM) trained on membrane protein sequences) |
| TMPRED  TMPRED | http://www.ch.embnet.org/software/TMPRED_form.html  Hofmann K, Stoffel W (1993) TMbase - a database of membrane spanning proteins segments. Biological Chemistry Hoppe-Seyler, 374, 166.  (uses statistical analysis of protein database) |
| TOPCONS  TOPCONS | http://topcons.cbr.su.se/  Bernsel A, Viklund H, Hennerdal A, Elofsson A (2009) TOPCONS: consensus prediction of membrane protein topology. Nucleic Acids Research, Web Server Issue 37, W465-W468.  (uses consensus of results of other prediction methods and sequence alignment of membrane protein sequences) |
| TOPCONS-single  TOPCONSs | http://single.topcons.net/  Hennerdal A, Elofsson A (2011) Rapid membrane protein topology prediction. Bioinformatics 27, 1322-1323. Bernsel A, Viklund H, Hennerdal A, Elofsson A (2009) TOPCONS: consensus prediction of membrane protein topology. Nucleic Acids Research, Web Server Issue 37, W465-W468.  (uses consensus of results of other prediction methods) |
| TOPPRED2  TOPPRED2 | ftp://ftp.pasteur.fr/pub/gensoft/projects/toppred/toppred-1.10.tar.gz  von Heijne G (1992) Membrane protein structure prediction, hydrophobicity analysis and the positive-inside rule. Journal of Molecular Biology, 225, 487-494. Claros MG, von Heijne G (1994) TopPred II: an improved software for membrane protein structure predictions. Comput Appl Biosci. 10, 6, 685-686.  (uses biophysical residue hydropathy values and other physico-chemical properties) |
| VALPRED  VALPRED | http://www.canoz.com/valpred/perl_valpred_2d.pl algorithm=VALPRED  (publication in preparation)  (uses threading and biophysical residue hydropathy values and solvent accessible surface area) |
| VALPRED2  VALPRED2 | http://www.canoz.com/valpred/perl_valpred_2d.pl algorithm=VALPRED2  (publication in preparation)  (uses threading and biophysical residue hydropathy values and solvent accessible surface area) |
| waveTM  waveTM | http://bioinformatics.biol.uoa.gr/waveTM  Pashou EE, Litou ZI, Liakopoulos TD, Hamodrakas SJ (2004) waveTM: wavelet-based transmembrane segment prediction. In Silico Biol, 4, 2, 127-131.  (uses biophysical residue hydropathy values and dynamic programming algorithm) |

***Supplementary Table S1B. Default benchmark parameters that can be adjusted by user.***

| **Parameter** | **Default Value** |
| --- | --- |
| Resolution and experimental method of 3D structures included in the benchmark standard | x-ray diffraction of resolution 3.5 Å or less, or NMR |
| Minimum length of observed and predicted helices (smaller helices are ignored) | 9 |
| Minimum overlap of observed helix residues for a helix prediction to score in the per-sequence-accuracy, per-segment-accuracy and average-helix-boundary-difference scores | 5 |
| Maximum distance in residues to include in the membrane-helix-boundary scores | 5 |
| Benchmark standard | OPM adjusted membrane helices |
| Homology level of transmembrane helix sequences | 30% similarity by global sequence alignment |
| Include sequences from β-barrel proteins | none |
| Include sequences from soluble proteins | none |

***Supplementary Table S2. Benchmark metrics and their formulae.***

| **Metric Abbreviation** | **Metric Name** |
| --- | --- |
|  | **Metric Definition** |
| TOPOGRAPHY SCORES (for scoring predictions of membrane helices versus not membrane helix) | |
| Per protein accuracy score : | |
| Qok% | Percentage of protein sequences for which all membrane helices are predicted correctly |
|  | number of protein sequences (chains) having  all observed membrane helices are predicted by the prediction method  and all predicted membrane helices are actually observed in the benchmark data  Qok% = __________________________________________________________________ x 100  number of protein sequences (chains) |
| Per segment accuracy scores : | |
| Qhtm %obs | Percentage of all observed membrane helices that are predicted correctly |
|  | number of membrane helices observed in the benchmark data  that the prediction method did predict as being membrane helices  Qhtm %obs = __________________________________________________________________ x 100  number of membrane helices observed in the benchmark data |
| Qhtm %prd | Percentage of all predicted membrane helices that are predicted correctly |
|  | number of membrane helices predicted by the prediction method  that are actually observed in the benchmark data as being membrane helices  Qhtm %prd = __________________________________________________________________ x 100  number of membrane helices predicted by the prediction method |
| Helix boundary accuracy scores : | |
| AvHb diff | Average helix boundary position difference in residues for the prediction versus the observed helix boundaries |
|  | ∑ \| ( distance in residues of observed helix boundary minus predicted helix boundary ) \|  AvHb diff = _________________________________________________________________________  number of membrane helices observed in the benchmark data x 2  that the prediction method did predict as being membrane helices  ( non-predicted observed helices and non-observed predicted helices are not included ) |
| QHb %obs | Percentage of all observed membrane helix boundaries (2 per helix) that are predicted correctly  (within distance from observed boundary) |
|  | number of membrane helix boundaries observed in the benchmark data  for which the prediction method predicted the helix boundary  within a certain number of residues from the boundary actually observed in the benchmark data  QHb %obs = ________________________________________________________________________________ x 100  number of membrane helices observed in the benchmark data x 2 |
| Gauss  QHb %obs | Scaled percentage of all observed membrane helix boundaries (2 per helix) that are predicted correctly  (score is scaled as a Gaussian curve normal distribution (mean = 0, variance = std-dev^2^ = 5 or chosen by user) around the observed helix boundary because observed helix boundary may not be exact) |
|  | ∑ ( score for each membrane helix boundary predicted by the prediction method )  Gauss QHb %obs = _________________________________________________________________________  ∑ ( score for each membrane helix boundary predicted by the prediction method )  Standard-deviation = Maximum distance in residues to include in the membrane-helix-boundary scores  Distance = predicted helix boundary – observed helix boundary  score for each membrane helix boundary = score-numerator _  predicted by the prediction method score-denominator  ( if predicted helix boundary is within or equal to 2 x standard-deviation from the observed helix boundary;  otherwise score = 0 )  Score-numerator = _ 1_________ x exp( - ( distance - mean )^2^ / (2 x standard-deviation ^2^ ) )  std-dev x √ ( 2 x Π )  Score-denominator = _ 1_ ______ x exp( - ( mean )^2^ / (2 x std-dev^2^ ) ) = 1  std-dev x √ ( 2 x Π ) std-dev x √ ( 2 x Π )  ( Thus, when predicted helix boundary is same as observed helix boundary,  then distance = 0, and score = 1 or 100% ) |
| Per residue accuracy scores : | |
| Q2% | Percentage of correctly predicted residues in two-states : membrane helix / not part of membrane helix  (each sequence contributes equally so that long sequences don't dominate the score) |
|  | Q2% = ( ∑ ( num residues in a seq that were correctly predicted as being membrane helix or not ) ) x 100 / number of sequences  number of residues in that sequence |
| htm MCC | Matthews Correlation Co-efficient^*^ for prediction of residues  as membrane helical vs. not membrane helical |
|  | (TP*TN) - (FP*FN) htm MCC = ________________________________________   √ ( (TP+FP) x (TP+FN) x (TN+FP) x (TN+FN) )  TP (true positives) = number of correctly predicted membrane helix residues TN (true negatives) = number of residues correctly predicted as not in a membrane helix FP (false positives) = number of incorrectly predicted membrane helix residues FN (false negatives) = number of residues incorrectly predicted as not in a membrane helix  ^*^Matthews B (1975) Comparison of the predicted and observed secondary structure of T4 phage lysozyme. Biochimica et Biophysica acta, 405, 442-451. |
| Q2T %obs | Percentage of all observed membrane helix residues that are predicted correctly |
|  | number of residues predicted as being part of a membrane helix  that really are observed in the benchmark data as being in a membrane helix Qhtm %obs = _________________________________________________________________________ x 100   number of residues that are observed in the benchmark data as being in a membrane helix |
| Q2T %prd | Percentage of all predicted membrane helix residues that are predicted correctly |
|  | number of residues predicted as being part of a membrane helix  that really are observed in the benchmark data as being in a membrane helix Qhtm %prd = _________________________________________________________________________ x 100   number of residues predicted as being part of a membrane helix |
| Q2N %obs | Percentage of all observed non-membrane helix residues that are predicted correctly |
|  | number of residues predicted as NOT being part of a membrane helix  that really are observed in the benchmark data as NOT being in a membrane helix Q2N %obs = ______________________________________________________________________________ x 100   number of residues that are observed in the benchmark data as NOT being in a membrane helix |
| Q2N %prd | Percentage of all predicted non-membrane helix residues that are predicted correctly |
|  | number of residues predicted as NOT being part of a membrane helix  that really are observed in the benchmark data as NOT being in a membrane helix Q2N %prd = ______________________________________________________________________________ x 100   number of residues predicted as NOT being in a membrane helix |
| TOPOLOGY SCORES (for scoring predictions of inside/outside topology) | |
| Per protein accuracy score : | |
| Qok3% | Percentage of protein sequences for which all inside, outside and membrane helix topologies are predicted correctly |
|  | number of protein sequences (chains) having Qok3% = all observed segment topologies (inside, outside or membrane helix) are predicted by the prediction method  and all predicted segment topologies are actually observed in the benchmark data  ________________________________________________________________________________ x 100   number of protein sequences (chains) |
| Nterm % | Percentage of protein sequences for which the topology of the N-terminal topology is predicted correctly |
|  | number of protein sequences (chains) having Nterm = N-terminal topology predicted correctly (inside or outside side of membrane)  ________________________________________________________________________________ x 100   number of protein sequences (chains) |
| Per segment accuracy scores : | |
| ioSeg Q2% | Percentage of correctly predicted topology segments in two-states :  inside side / outside side of membrane  (each sequence contributes equally so that a topology prediction that starts correctly and finishes incorrectly  due to a missed membrane segment prediction rather than due to an incorrect topology prediction start  will be penalised as an incompletely correct topology prediction (or not completely incorrect prediction) and long sequences will not be overly penalised in the final score) |
|  | number of correctly predicted non-membrane topologies  ioSeg Q2% = ( ∑ ( ( inside side or outside side of membrane) predicted in a sequence ) ) x 100 / num seqs  num non-membrane topologies  (inside side or outside side of membrane) observed in that seq |
| Qiom %obs | Percentage of all observed topologies (inside, outside or membrane helix) that are predicted correctly |
|  | number of topology segments (inside, outside or membrane helix) observed in the benchmark data Qiom %obs = that the prediction method did predict as being the correct type of topology  ________________________________________________________________________________ x 100   number of topology segments observed in the benchmark data |
| Qio %obs | Percentage of all observed non-membrane topologies (inside side or outside side of membrane) that are predicted correctly |
|  | number of non-membrane topologies (inside side or outside side of membrane)  observed in the benchmark data Qio %obs = that the prediction method did predict as being the correct type of non-membrane topology  ________________________________________________________________________________ x 100   number of non-membrane topologies observed in the benchmark data |
| Per residue accuracy scores : | |
| Q3% | Percentage of correctly predicted residues in three-states :  membrane helix / inside non-membrane residue / outside non-membrane residue  (each sequence contributes equally so that long sequences don't dominate the score) |
|  | number of residues in a sequence that were correctly predicted as being  Q3% = ( ∑ ( membrane helix, inside non-mumbrane residue or outside non-membrane residue ) ) x 100 / number of sequences  number of residues in that sequence |
| ioRes Q2% | Percentage of correctly predicted residues in two-states :  inside non-membrane residue / outside non-membrane residue  (each sequence contributes equally so that long sequences don't dominate the score) |
|  | number of non-membrane residues in a sequence  that were correctly predicted as being  ioSeg Q2% = ( ∑ ( ( inside non-membrane or outside non-membrane membrane residue ) ) x 100 / number of sequences  number of non-membrane residues in that sequence |
| io MCC | Matthews Correlation Co-efficient^*^ for topology prediction of residues  as either on the inside vs. the outside side of the membrane |
|  | (TP*TN) - (FP*FN) io MCC = ________________________________________   √ ( (TP+FP) x (TP+FN) x (TN+FP) x (TN+FN) )  TP (true positives) = number of non-membrane residues on the inside side of the membrane that are correctly predicted TN (true negatives) = number of non-membrane residues on the inside side of the membrane FP (false positives) = number of non-membrane residues on the outside side of the membrane that are correctly predicted FN (false negatives) = number of non-membrane residues on the outside side of the membrane  ^*^Matthews B (1975) Comparison of the predicted and observed secondary structure of T4 phage lysozyme. Biochimica et Biophysica acta, 405, 442-451. |

Inside refers to the inside side of the membrane, and outside refers to the outside side of the membrane.
